# Encoding of Race Categories by Single Neurons in the Human Brain

**DOI:** 10.3390/neurosci3030031

**Published:** 2022-08-05

**Authors:** André B. Valdez, Megan H. Papesh, David M. Treiman, Stephen D. Goldinger, Peter N. Steinmetz

**Affiliations:** 1 Neurtex Brain Research Institute, 8300 Douglas, Suite 800, Dallas, TX 75225, USA; 2 Department of Psychology, New Mexico State University, Las Cruces, NM 88003, USA; 3 Department of Neurology, Barrow Neurological Institute, Phoenix, AZ 85013, USA; 4 Department of Psychology, Arizona State University, Tempe, AZ 85287, USA

**Keywords:** human single neuron, hippocampus, amygdala, human race perception

## Abstract

Previous research has suggested that race-specific features are automatically processed during face perception, often with out-group faces treated categorically. Functional imaging has illuminated the hemodynamic correlates of this process, with fewer studies examining single-neuron responses. In the present experiment, epilepsy patients undergoing microwire recordings in preparation for surgical treatment were shown realistic computer-generated human faces, which they classified according to the emotional expression shown. Racial categories of the stimulus faces varied independently of the emotion shown, being irrelevant to the patients’ primary task. Nevertheless, we observed race-driven changes in neural firing rates in the amygdala, anterior cingulate cortex, and hippocampus. These responses were broadly distributed, with the firing rates of 28% of recorded neurons in the amygdala and 45% in the anterior cingulate cortex predicting one or more racial categories. Nearly equal proportions of neurons responded to White and Black faces (24% vs. 22% in the amygdala and 26% vs. 28% in the anterior cingulate cortex). A smaller fraction (12%) of race-responsive neurons in the hippocampus predicted only White faces. Our results imply a distributed representation of race in brain areas involved in affective judgments, decision making, and memory. They also support the hypothesis that race-specific cues are perceptually coded even when those cues are task-irrelevant.

## 1. Introduction

Group membership can be based on many attributes, including race and affects brain activity involved in different types of perception (e.g., related to words, actions, and faces [1])(There is a current debate over whether “race” or “ethnicity” should be used to refer to the phenotypic characteristics that are varied in this experiment. We use the term “race” to refer to such differences, remaining consistent with much of the previous neuroimaging literature.). Multiple studies in neuroscience have shown that people process cues about in-group vs out-group members differently [2,3,4,5], which may correlate with biased perceptions, attitudes, and behaviors. 

Studies using functional magnetic resonance imaging (fMRI) have shown that brain areas involved in face processing produce less hemodynamic activation to out-group faces [6], while the amygdala (which is involved in the perception of face gaze, affect, and familiarity [7,8,9,10,11,12,13]) produces early [14] and sustained [15] hemodynamic changes in response to out-group faces. Other brain areas, like the prefrontal cortex, play a role in accessing social knowledge [16] and may regulate race evaluation [14]. For example, there is increased frontal activity for remembered in-group faces early during memory retrieval [17]. Functional imaging has further shown that activity in the anterior cingulate cortex (ACC), which is involved in decision making and affect, tracks out-group versus in-group trust [18], reflects the observation of in-group non-verbal actions [19], and varies when a person observes painful stimulation applied to in-group versus out-group people [20].

Imaging studies have thus provided evidence, via changes in regional blood flow, regarding the neural correlates of racial processing in face perception. Collectively, these studies tend to show different hemodynamic responses (whether excitatory or inhibitory) to racial in-group versus out-group people across tasks (e.g., face recognition and classification, observation of social interactions, and observation of applied stimulation to faces). By contrast, the present investigation tested whether single-neuron firing rates are sensitive to variations in race features during a face classification procedure. Several previous single-neuron recording studies have focused on the processing of emotion in facial expressions [21,22,23,24] and gender and affect [25]. However, due to the scarcity of unit-level studies of racial and other social cues [21,22] and inconsistencies in fMRI findings e.g., out-group-elicited bilateral amygdala activity in some cases [15,26] and right-lateralized activity in others [14], we sought to gather more direct evidence of neural processing in race perception. 

The present study was designed as an initial exploratory study of how the activity of single neurons in the human brain change their firing rate depending on unattended perceptual cues of race. In this study, subjects viewed realistic synthetic faces which were varied in both race and the emotion depicted. Subjects classified the faces as having a positive or negative expression and variations in racial features were irrelevant to the patients’ task and were orthogonal to the depicted expressions. The volunteers were patients with epilepsy, undergoing clinical microwire recording to locate and surgically assess seizure onset zones. In these patients, we recorded neurons in the amygdala, ACC, hippocampus, and ventromedial prefrontal cortex (vmPFC). How neural firing rates in this experiment reflect the gender of the participants has previously been reported [27]. This paper reports how the firing rates depend on the race of the presented face. 

Given the hypothesis that race is perceptually processed as an automatic, categorical response [28], we predicted that we would observe race-selective neural firing, despite attention being oriented toward an orthogonal stimulus dimension. In addition, we examined whether neurons in these brain areas would show in-group or out-group selectivity, as observed in fMRI, which could suggest a potential correlation between unit-level coding and responses in regional blood flow [25,29,30]. 

To anticipate our results, we indeed observed race-signaling neurons in the hippocampus, ACC, and amygdala, but not the vmPFC. Thus, despite race variations being task-irrelevant, notable proportions of recorded neurons responded in a race-selective manner. Regression models showed that neural firing rates in the amygdala and ACC predicted race categories in a distributed fashion, i.e., with apparently broad tuning for several race categories and low lifetime sparsity, or a large proportion of stimuli eliciting a significant response across the brain areas [31]. In addition, nearly equal proportions of neurons in the amygdala and ACC responded to white and black faces, in contrast to the outgroup-specific responses in these brain areas reported in the fMRI literature. Our results suggest that neurons were broadly sensitive to perceptual features indicating race, rather than being highly selective for either in-groups or out-groups. 

## 2. Method

**Participants and Exclusions.** We recorded single-neuron activity from 14 patients at the Barrow Neurological Institute. The original sample included 10 Caucasian White, two Caucasian Hispanic, and two African-American Black patients (see further details in Appendix A). All patients had drug-resistant epilepsy and were being evaluated via microwire recording for possible resection of epileptogenic foci. Each patient consented both verbally and in writing to participate per a protocol approved by the Institutional Review Board of Saint Joseph’s Hospital and Medical Center in October 2005, August 2007, May 2009, November 2010, October 2012, and February 2013. The patients were invited to complete multiple experimental sessions. Since microwires shift slightly as patients spend time in the hospital, repeated sessions were treated as independent samples (i.e., recordings from different neurons). The number of patients was determined by clinical scheduling and recordings were continued until more than 400 single unit recordings had been obtained. 

During the 7–10 days that patients were in the Epilepsy Monitoring Unit and performing these experiments, they were gradually tapered off their anti-epileptic medications in order to increase the probability of them having seizures. It is thus possible that these medications may have affected their cognitive performance on the experimental tasks. 

In cognitive experiments on race perception, researchers typically strive to recruit near-equal participant groups matching the racial backgrounds depicted in the stimulus faces (e.g., [32]). Given our patient population, such balanced sampling was not practical: the Barrow Neurological Institute (BNI) only evaluates 8–10 microwire patients per year and fewer than 6% of Arizona residents are African-American, making it nearly impossible to collect a robust African-American sample. To guide our approach, we conducted initial analyses, comparing White and Black participants with respect to fractions of neurons per brain area that differentially responded, based on stimulus race. This analysis involved first performing one-way ANOVAs for an effect of stimulus race on the response of individual neurons. Next, the fractions of neurons in each brain area with a significant response were compared between subject races using Fisher’s Exact Test for fractions [33]. This test showed no significant interaction between subject and stimulus races, perhaps reflecting the small African-American sample. For maximum clarity, data analyses reported in the main text exclude the two African-American patients, allowing a clear division of “in-group” and “out-group” stimulus faces for the remaining participants. Results including all participants appear in Appendix B and were quite similar to the results presented in the main text. Finally, we excluded data from one patient, and two sessions from other patients, because of technical errors. Following exclusions, the sample size for all analyses was 11 patients, although our analyses treat recorded neurons as the fundamental unit of analysis. In an earlier report, Newhoff et al. [27] published results based on these data (focusing on facial affect), without excluding any patients or sessions. Our current focus on race perception required more stringent inclusion criteria.

**Microwire Bundles and Recording System.** Extracellular action potentials corresponding to single-neuron activity were recorded from microwire tips implanted bilaterally along with clinical depth electrodes used to record clinical field potentials [34,35]. The implantation sites were chosen according to clinical criteria, which limits the potential recording sites. For these patients, and per standard clinical practice at the BNI, the sites included the bilateral hippocampus, prefrontal cortex, anterior cingulate cortex, and amygdala. In the hippocampus, the wires were targeted to the midbody of the hippocampus, just behind the head of the hippocampus, opposite the apex of the cerebral peduncle. In the prefrontal cortex, the wires were targeted to the ventromedial prefrontal cortex, below the anterior cingulate gyrus. In the anterior cingulate, the wires were targeted to parts above and behind the genu of the corpus callosum. In the amygdala, the wires were targeted to be in the center of that structure.

Each recording site received a bundle of nine 38 µm-diameter platinum-iridium microwires (California Fine Wire, Grover Beach, CA, USA), implanted stereotactically (Medtronic StealthStation, Minneapolis, MN, USA) using a 1.5-T structural MRI. Each microwire typically had an impedance of 300 kΩ at 1000 Hz. Electrodes were placed through a skull bolt with a custom frame to align the depth electrode along the chosen trajectory. The error in tip placement using this technique is estimated to be ±3 mm based on manual inspection of the pre-operative MRI and post-operative CT and prior work [36,37]. While this technique, lacking co-registration of a post-operative CT with the preoperative targets, cannot guarantee placement within the targeted brain structures, the majority of microwire bundles are estimated to be in the targeted structures. We, therefore, refer to the position of the microwires as within their targeted structures throughout the results presented here; we address this limitation in the General Discussion.

The system used to record extracellular potentials from microwires has been previously described [38]. In brief, each bundle of nine microwires was attached to a custom-designed head-stage amplifier which provided preconditioning with a high pass filter (0.5 Hz), followed by a 10 kHz antialiasing filter and a computer-controlled 1–16× adjustable gain amplifier of the difference between eight of the microwires and a 9th microwire used as reference. The head-stage amplifiers were connected to custom-designed signal conditioning electronics and Data Translation A/D convertors (DT9834) digitized the signal at a sampling rate of 29,412 Hz. The digitized signals were recorded on a laptop computer (IBM Thinkpad, Armonk, NY, USA) for subsequent analysis using a custom C++ program.

**Figure 1:**
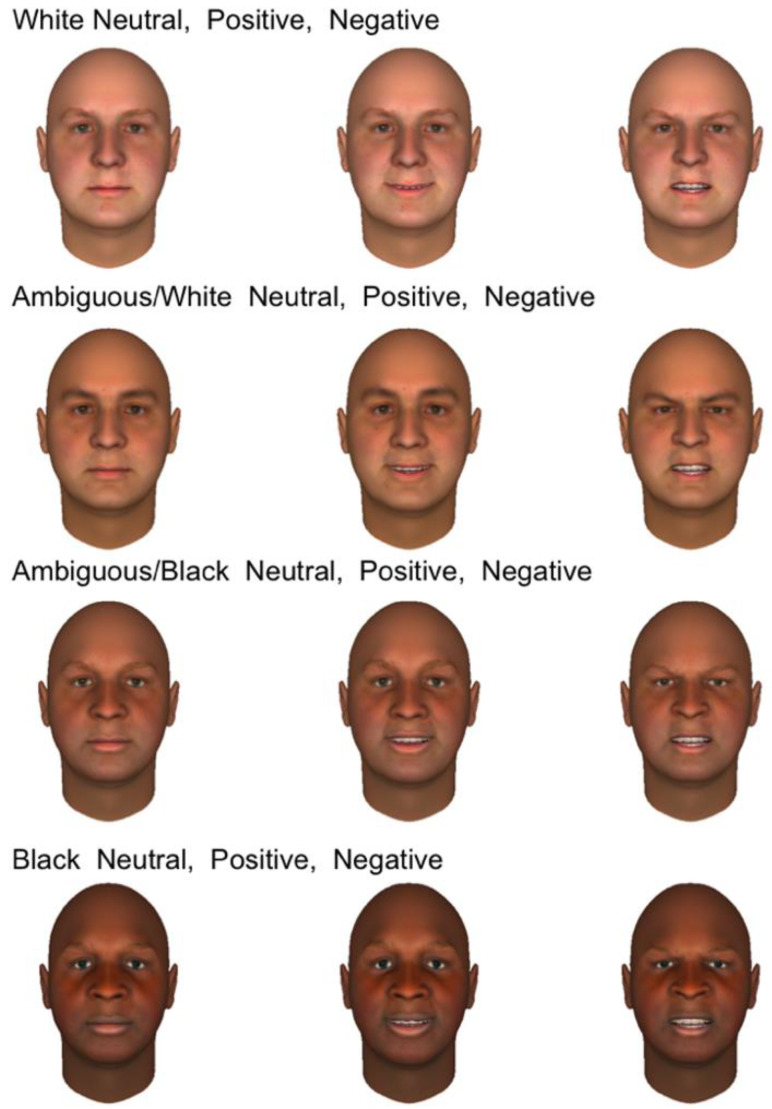
An example set of 12 FaceGen faces created from an original photograph. From left to right, columns show faces with neutral, positive, and negative expressions. With affect parameters frozen, faces were morphed along the race dimension, which involved simultaneous changes in both skin tone and facial morphology. From top to bottom, faces ranged from White to Black, with intermediate ambiguous versions.

**Stimuli and Validation.** Participants were asked to quickly classify the emotional expressions of synthetic male faces, created using FaceGen Modeler [39], a computer program that generates realistic synthetic faces from imported photographs. We selected original photographs from PAL and Colour FERET databases [40,41]. From a set of 20 White male faces, we generated computerized models equated for size and brightness, all with identical white backgrounds. Once a face model was created, it could be manipulated for emotional expression (using up to 40 parameters that modify the face asymmetrically) and morphed to create racial continua from White to Black. In this manner, we initially created 20 sets of 12 faces; an example set is shown in Figure 1. For each face, we first created subjectively positive (happy), negative (angry), and neutral versions, using different parameter combinations per set to allow for natural variation. After facial expressions were set, those relevant parameter values were frozen, and faces were morphed in four steps from “clearly White” to “clearly Black” (with “ambiguous” values in-between). For this initial study, the morphing process combined changes in skin tone and facial morphology, consistent with different ethnic backgrounds. Thus, this study cannot separate the effects of skin tone and facial morphology on the neural responses. For each affective expression, the same parameter values were selected to represent skin tone and morphological features. In total, participants viewed 120 faces per session (60 ambiguous, 30 Black, 30 White). We created two sets of 120 faces which could be used in separate sessions.

In the interest of clarity, we apply different terminology when referring to human participants versus computerized faces and response labels. When referring to experimental volunteers, we use the terms Caucasian and African-American. When referring to computerized faces or response options, we use the labels White and Black.

Since we created faces using subjective judgment, we validated the affect and race dimensions in two pilot experiments with Arizona State University students as volunteers. All self-reported having a normal or corrected-to-normal vision. In the first validation test, participants (*n* = 25) were shown all faces in random order, quickly classifying each as “positive” or “negative” in facial expression, with no option to respond “neutral.” Variations in the apparent race were included but were orthogonal to the task. “Positive” faces were classified as positive in 93.2% of trials (mean correct RT = 515 ms). “Negative” faces were classified as negative in 98.1% of trials (mean correct RT = 499 ms). “Neutral” faces were classified as positive in 41.5% of trials (mean RT = 688 ms) and as negative in 58.5% of trials (mean RT = 601 ms). The results verified that positive and negative expressions were classified quickly and accurately, and that neutral faces were more challenging. There was also a slight bias toward interpreting neutral expressions as being negative.

To validate the race dimension, we asked 33 undergraduate volunteers to quickly classify each stimulus face as “White” or “Black,” with no option to respond “ambiguous.” Participants were instructed to ignore variations in affective expressions. Classification agreement was high, and RTs were short when the non-ambiguous faces were classified: White faces were correctly classified in 98.9% of trials (RT = 544 ms) and Black faces were correctly classified in 97.8% of trials (RT = 518 ms). Among the intermediate items, ambiguous White faces were predominantly classified as White (73.4% of trials; RT = 649 ms) but were occasionally classified as Black (26.3% of trials; RT = 712 ms). The ambiguous Black faces were predominantly classified as Black (78.3% of trials; RT = 610 ms) but were occasionally classified as White (21.7% of trials; RT = 709 ms). Thus, classification decisions generally followed the intended categories, with slower RTs to ambiguous faces.

**Figure 2:**
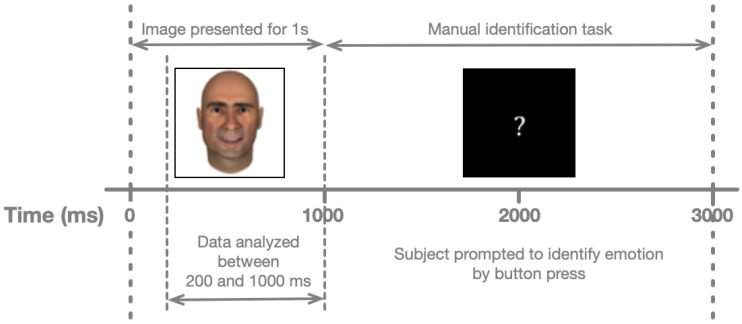
Schematic trial structure. The horizontal axis represents the passage of time in milliseconds. Dashed lines indicate key stages per trial and labeled arrow bars distinguish between the recording and behavioral segments. From Newhoff et al., 2015, Figure 2.

**Experimental Procedure.** In testing sessions with the hospital patients, face images were presented using a laptop computer (Apple Computer, Cupertino, CA, USA) with a 15-inch screen. Each patient was seated in a hospital bed, with the laptop placed on an overbed table. Face images occupied a square of 0.1 m on each side. At a distance of ~0.5 m, this area subtends ~11° of visual arc. Responses were collected from a trackball with large buttons (ExpertMouse, Kensington, Redwood Shores, CA, USA) to increase participant comfort and provide electrical isolation from the laptop switching power supply. As shown in Figure 2, each face was shown for one second, including the relevant analysis period (200–1000 ms after stimulus onset). Once the face disappeared, participants had two seconds to classify its affect. Stimulus presentation, response collection, and synchronization with the neural recordings were performed using a JAVA (Sun Microsystems, Santa Clara, CA, USA) program developed by our laboratory. Each face was presented six times, for a total of 720 trials.

**Spike Sorting and Neuronal Responses.** In total, we recorded from 1024 channels in 25 experimental sessions. None of the sessions were recorded while patients experienced seizures. Analyzed recording channels included those from brain areas subsequently identified as having clinical seizure foci, as well as those without. We used established spike-sorting methods [38] to isolate single-neuron activity in each recording. In brief, possible action potentials (events) were detected by filtering with a bandpass filter, 300–3000 Hz, followed by a two-sided threshold detector (threshold = 2.8 times each channel’s standard deviation) to identify event times. Event waveform shapes surrounding each event were extracted with the event time aligned at the 9th of 32 samples. All events captured from a particular channel were separated into groups of similar waveform shapes (clusters) using the open-source clustering program, KlustaKwik (http://klustakwik.sourceforge.net, accessed on 30 August 2012), which applies a modified version of the Govaert-Celeux expectation maximization algorithm [42,43]. The first principal component of all event shapes recorded from a channel was the waveform feature used for sorting. After sorting, each cluster was classified as being noise, multi-unit activity (MUA), or single-unit activity (SUA), using the criteria described in (Valdez et al. [44], Table 2). From 1024 recorded channels, we isolated 881 clusters of SUA. There were 625 clusters of SUA in the non-excluded sessions for the primary results reported here. In agreement with prior studies [27,45,46], we used the number of sorted action potentials in an interval spanning 200–1000 ms after stimulus onset as our dependent measure of neuronal responses to each stimulus. Per consensus in the field, we considered recordings from the same channel in separate sessions to be independent observations. To further check that this treatment is correct, we identified all clusters of SUA recorded on the same recording channel in multiple sessions which had average firing rates differing by less than 50% between sessions and waveform shapes that retained the same sign (positive or negative peak) between sessions. There were 19 such clusters in total. After eliminating these from the dataset, we obtained the same results as reported here with minor variations. 

**Multinomial Logistic Regression.** In order to determine whether neuronal firing in the response interval (200–1000 ms after stimulus onset) changed from background firing in a manner that indicates the race of face presented (stimulus race), we used logistic regression with the ratio of the response firing rate on each trial compared to the firing in a background interval of the same length prior to stimulus onset (1000–200 ms before stimulus onset; [44,47,48]). Our sole predictor (the number of action potentials within the 200–1000 ms window) was assigned a coefficient, indicating the degree of the predictor’s correlation with the predicted outcomes. Statistically significant changes in coefficient values from 0 (i.e., excitatory or inhibitory differences in firing rates, measured by *t*-tests for nonzero coefficients) could signal different odds of any race category being present, versus a reference level (background firing rate), where odds ratios reflect the probability of a category is present, given a unit change in the predictor [49]. This is a simplified version of the point-process framework that Truccolo et al. [50] proposed.

After determining the coefficients for neurons, we compared the numbers of neurons in each brain area that significantly predicted race categories to those expected by chance under a binomial distribution, using a Benjamini–Hochberg adjustment for false discovery rate [51]. All statistical tests used a significance criterion of 0.05 and were performed using the R statistical package [52].

**Changes from Background Firing.** The multinomial logistic regression tests inherently compare the response to background firing; however, to increase confidence that the observed neuronal responses reflected stimulus-driven activity, rather than noise, we also used a hypothesis test to determine how probable the observed responses would be under a combined null hypothesis. This null hypothesis was that firing rates were equivalent for all race categories (equivalent to a one-way ANOVA) and were also equal to their respective background firing rates [53]. We applied a resampling technique [54] wherein we computed the empirical distribution of a test statistic equal to the sum of the squared deviations of the mean responses for each race category from the mean background firing rate. We then computed the probability of obtaining the observed test values. This test indicates how unlikely it would be to observe the responses, divided among race categories if neural firing rates were insensitive to such information. 

**Information To Decode Race Categories.** In order to further examine the ability to predict stimulus race based on neural firing, we computed the mutual information between neural response counts (in the 200–1000 ms interval) and stimulus races for all trials, in each brain region [52, infotheo package, mutinformation function using the entropy measure]. 

## 3. Results

The primary questions we sought to answer in this initial study were (1) whether individual neurons would preferentially respond to certain race categories, (2) whether such responding would vary according to brain regions, (3) whether stronger responses would be observed for either black or white faces, and (4) whether neurons would appear coded for specific races or would be more generally sensitive to changes across trials. 

**Neuronal Firing Rates Predict Race Categories.** We first applied multinomial logistic regression models to predict, based on neural firing rates, when patients were viewing specific race categories. In this primary analysis, we report results only from single-unit activity (SUA), or well-isolated neurons, bilaterally by brain area among 11 Caucasian participants. Appendix B shows results across all enrolled volunteers (including two African-American patients) and also results from multi-unit activity (MUA).

**Figure 3:**
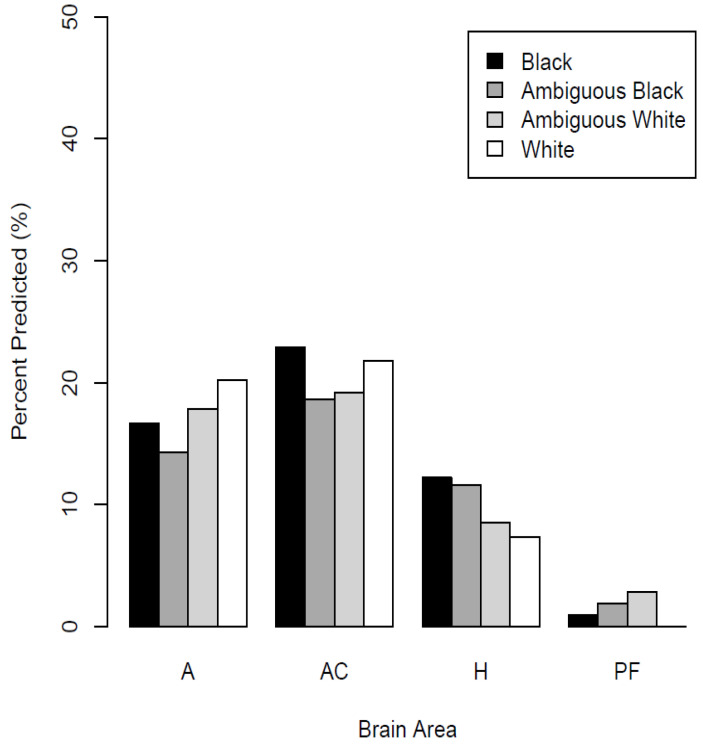
Percentages of neurons (SUA) significant for a test of the effect of stimulus race category on firing rate (relative to background firing) by brain area. The *x*-axis shows brain areas. The total neurons recorded were: Amygdala (*n* = 168), ACC (*n* = 188), Hippocampus (*n* = 164), and the percentages stated are the corresponding percentages of these totals in each brain area. Note that there was no significant percentage of race-predictive neurons in the vmPFC out of the total (*n* = 105).

Figure 3 shows the percentage of neurons in each brain area that significantly predicted each race category. These results are derived across all recordings, calculated using the number of well-isolated neurons in each brain structure as denominators, thus no error bars appear in Figure 3. In the amygdala, ACC, and hippocampus, there were significant numbers of neurons that predicted the depicted race categories (binomial test, *p* < 0.05). In the vmPFC, the percentage did not reliably differ from zero (*p* > 0.05). The percentages for each brain area and racial grouping are listed in Table 1. 

When doing so, there were no apparent differences in the percentages of neurons predicting racial category in the amygdala (24% vs. 22%) or ACC (26% vs. 28%). However, there was a more notable difference in the percentages of hippocampal neurons predicting racial category (12% vs. 19%).

**Figure 4:**
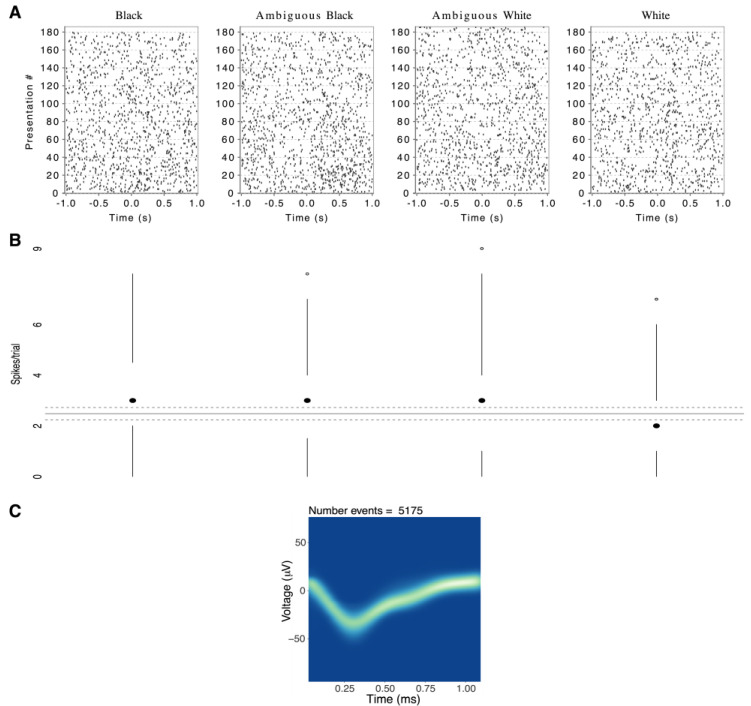
Responses of a single neuron in the left amygdala of one subject, predictive of the black and ambiguous black stimulus categories. (**A**) Raster plots of firing for each presentation of a face from each race category. Time 0 is the onset of stimulus presentation. (**B**) Modified boxplot of firing rates for all presentations of a face from each race category. The center dots show the median response per race category. Vertical lines extend from ±(1.58*IQR)/sqrt(*n*), where IQR = interquartile range, *n* = number of observations. This is equivalent to a 95% confidence interval for differences between medians. Small circles show responses outside that range. The solid grey line shows the mean of background firing; dashed grey lines represent a 95% confidence interval for the median of background firing. (**C**) Density plot of the extracellular potential waveforms for each putative action potential from this neuron. Threshold crossing at 0.27 ms.

To illustrate the magnitude of a significant response to one race category, relative to background firing rate, Figure 4 shows an elevated median response from one left-amygdala neuron in response to Black and Ambiguous Black faces. Overall, while response magnitudes were low in terms of absolute spikes per trial, they were comparable to prior human single-neuron studies [27,44,45,55].

**Changes from Background Test (CBT).** As illustrated by one neuron in Figure 4, even when neural firing predicted race categories, the response differences were often subtle. This may reflect our experimental procedure, which required focused attention to emotional expressions, whereas race varied as an irrelevant background dimension. To further test that the observed responses were not due to random fluctuations of background activity, we used a bootstrapping procedure as described in Methods [53].

Table 2 shows there were significant differences between responses and background counts in more neurons than expected by chance (*p* < 0.05) in all brain areas. When these significant neural responses were further subdivided by race categories, the results were virtually identical to those in Figure 3, suggesting that the logistic regression and bootstrapping analyses produced a very high agreement.

**Figure 5:**
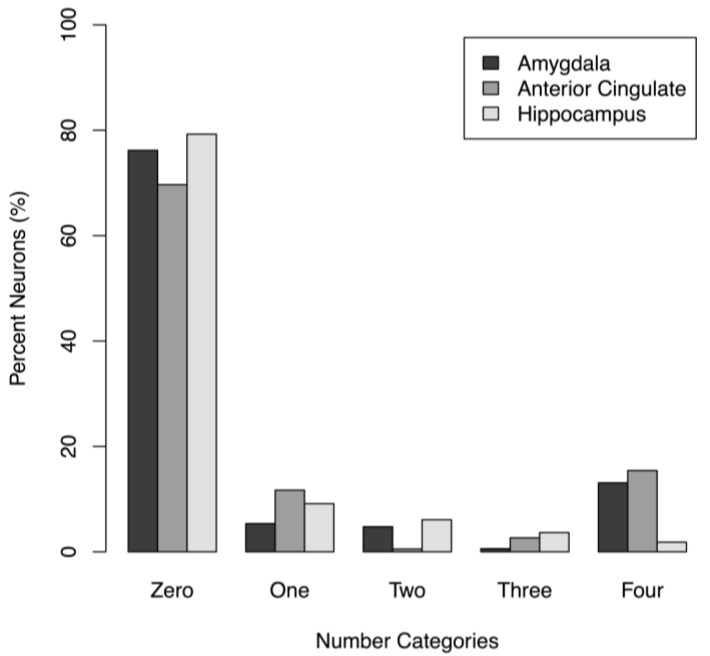
Percentages of neurons that significantly coded different numbers of race categories. The *x*-axis shows the number of race categories observed per neuron; bar shading indicates brain areas. Amygdala (*n* = 168), ACC (*n* = 188), Hippocampus (*n* = 164). There were no significant proportions of race-predictive neurons in the vmPFC (*n* = 105).

**Number of Race Categories Encoded.** To expand upon the results in Figure 3, we next tested whether race-responsive neurons were selective (e.g., preferentially responding to one specific race) or were more broadly tuned. For each brain area, we counted neurons that significantly coded zero, one, two, three, or four race categories in the multinomial logistic regression analysis. Coding four race categories, for example, suggests general responsiveness to all presented images. The results are shown in Figure 5. In the amygdala, 5% of neurons encoded a single race category, while 18% of neurons encoded two or more categories. In the ACC, 12% of neurons encoded a single category, while 19% encoded two or more categories. In the hippocampus, 9% encoded a single category, while 12% encoded two or more categories. As shown, roughly 60% of all recorded neurons showed no reliable firing-rate changes based on stimulus race. 

**Calculation of information to decode race categories.** As shown in Figure 5, neural responses to race categories were rarely one-to-one, we found robust differences in the average amounts of available information across brain areas (ANOVA, *p* = 0.007). Table 3 shows the amount of information available per brain area, along with the neurons per area that would be required to distinguish among all four categories (i.e., two bits of information). As shown, slightly fewer neurons are required in the amygdala (relative to other brain areas) to successfully decode stimulus races, whereas considerably more neurons are required in the ventromedial prefrontal cortex. The latter result is not surprising, as the vmPFC showed relatively few race-sensitive neurons. 

As previously reported, there were robust effects of the depicted emotions [27], which directly occupied participants’ attention.

## 4. Discussion

The present study examined single-neuron responses during perception of Black and White faces, collected during a task wherein facial emotions were classified, and the race was varied as an orthogonal, irrelevant dimension. Despite the attention being directed toward facial expressions, we observed race-selective neural firing rates in the amygdala, ACC, and hippocampus. Regression models showed notable percentages of neurons encoded multiple race categories in the amygdala and ACC, but not the hippocampus. Separate tests verified that race-predictive neurons had firing rates that reliably differed from background firing upon stimulus presentation. The tuning of race-predictive neurons in the amygdala and ACC appears rather broad, characterized by responses to one or more race categories, with low lifetime sparsity [56]. This type of sparsity is defined as the fraction of stimuli that evoke statistically reliable neuronal responses [31]. Although our main analyses included only Caucasian participants viewing White and Black faces, the results imply a distributed neural representation for racial categories. This is generally consistent with Valdez et al.’s study [44] showing distributed coding (with similarly broad tuning and low lifetime sparsity) for stimuli from several categories, including faces, during a similar visual discrimination task. Tuning in the hippocampus showed lower lifetime sparsity, with a greater fraction of neurons responding to zero or just one race category.

Our results showed that similar percentages of neurons in the amygdala and ACC predicted the presence of racial groups, a result at odds with prior reports in the imaging literature. fMRI studies have suggested different hemodynamic responses when people perceive in-group vs out-group members [57,58,59,60]. In the amygdala, for example, there is greater activation when subjects briefly view racial out-group faces, while in the prefrontal cortex, there is greater activation when the same subjects view racial in-group faces [14]. Racial out-group-related increases in amygdala activity also occur when subjects perform social categorization based on perceived face ages [61]. Such differential activation regarding racial in-group versus out-group also occurs in the ACC across various tasks [1].

By contrast, we did not find neurons in the amygdala or ACC that were disproportionately selective for either racial group presented. This seems to indicate a lack of correlation between single-neuron coding and race-driven changes in regional blood flow. Such a disparity may arise because we did not record local field potentials (LFPs), a peri-synaptic activity thought to be comprised of excitatory and inhibitory postsynaptic potentials and dendritic afterhyperpolarizations, which have a known correlation with blood oxygen level-dependent (BOLD) signal changes [62]. The disparity could also reflect the narrow spatial focus of a human single-neuron recording or it could confirm a reported dissociation between BOLD signals, spiking activity, and LFPs [63]. Task differences may also help explain the different neural responses. Cunningham et al. [14] observed race-selective BOLD differences in the amygdala only when faces were shown very briefly (30 ms), not with longer exposures (525 ms). In the current study, faces were shown for 1000 ms, well beyond the race-selective “window” suggested by the Cunningham et al. results. Additionally, neural responses to race features were expected to be somewhat attenuated in the present study, as attention was task-oriented toward the orthogonal emotional dimension. Clearly, more data from these different methodologies would allow stronger conclusions about neural responses to racial features, as well as verifying the out-group selectivity we observed in hippocampal neurons.

The patients in this experiment have epilepsy, which may broadly affect their cognition. Additionally, we specifically recorded brain areas that were identified as potentially containing seizure onset zones. Although prior research suggests that epilepsy does not affect neural firing patterns in response to visual stimuli [64], we re-analyzed our recordings for responses to racial categories, excluding any areas that were identified as potential seizure onset zones. The results, presented in Appendix B, confirmed the existence of broadly distributed race representation, as in Figure 3 and Table 1, and also showed a significant fraction of neurons responding to the ambiguous in-group in the hippocampus. As noted earlier, our participants were limited to patients receiving microwire evaluation, preventing us from achieving a balanced design with respect to the patients’ races. With only two African-American participants, we could not properly test a full 2 × 2 design, crossing subject, and stimulus races. We, therefore, excluded those two participants in the results presented above but did analyze their results, as summarized in Appendix B. The analyses showed no effects related to the participant’s race. Future studies to examine this question in greater detail could include a larger number of African-American participants or could analyze data averaged over many neurons. 

Another future question building on these results is the relative contributions of skin tone versus facial morphology to the changes in neural firing rates reported here. The stimuli used combined changes in skin tone and facial morphology when varying race. Since we previously described changes in firing rates in these brain areas depending on the brightness and contrast of images [65], it would be informative to vary skin tone and facial morphology and determine how each contributes to variations of firing rate with race. In the current study, however, we expected neural responses to racial variations to be relatively attenuated, as attention was selectively directed to facial affect. Given prior research in face perception [28,66,67], it is likely that skin color is the most diagnostic stimulus dimension during rapid face classification. For our purposes, spiking rates suggested that MTL neurons track humans’ implicit classification of faces by race, despite such variations being task irrelevant. Even if the neural responses we observed were driven by skin color variations, they illustrate how the brain responds to socially relevant facial features, regardless of selective attention being focused elsewhere (see also [68] for classic research on dimensional interactions during stimulus processing).

Since the recording methods employed in this study did not allow the determination of sub-nuclei within the various brain areas, future studies could employ more accurate localization to discern potential functional differences within separate nuclei of the amygdala. For example, such a study could examine differences in neuronal responses in the basolateral and lateral amygdala when processing fearful and reward-related stimuli [69]. 

Although our results did not reveal strong selectivity for either racial group in brain areas critical for social judgments and decision making, they do constitute further evidence for neural representations of race that carry several implications. First, the results support the hypothesis that race is processed as a basic perceptual feature [67], triggering robust neural responses even when attention is focused on an orthogonal stimulus dimension (in this case, facial affect). As noted, prominent theories of the other-race effect posit that the human brain classifies racial outgroup members categorically, only further processing them as individuals with extended effort [28]. Such differences in immediate perception are not universal, for example being absent when people perform match-to-sample tasks with in-group and out-group faces [70,71]. Notably, the match-to-sample task requires focused attention on precise facial features. When task performance does not demand face individuation, neural responses (such as repetition suppression in the FFA) suggest more categorical processing for out-group faces. Second, regarding specific brain structures, neural firing in the amygdala links stimulus perception to judgments [72,73]. As such, race responsiveness may represent a correlate of increased vigilance [74] (e.g., in dorsal subregions) and is evidence of sustained stimulus evaluation [75]. Race-selective neural firing in the ACC may signal preconscious race classification [18,19]. Activity in the ACC also likely reflected subjects’ sustained error monitoring during the experimental task.

The present study suggests that the perception of racial features has an identifiable and mostly distributed (rather than highly selective) neural basis. Other interesting lines of future research include whether race-specific neural firing changes after perceptual training [76], when faces are known individuals [77], or by examining perception in cultures that place less emphasis on dichotomized racial boundaries [78].

## Figures and Tables

**Table 1:** Percentages of neurons with a significant response to stimulus race category (relative to background firing rates) for each brain area. Asterisks denote significant proportions (*p* < 0.05). Percentages are the number of neurons with a significant test in each area divided by the total number in the area multiplied by 100.

	Number of Neurons	Black	Ambiguous Black	Ambiguous White	White
Amygdala	168	28 (17%) *	24 (14%) *	30 (18%) *	34 (20%) *
ACC	188	43 (23%) *	35 (19%) *	36 (19%) *	42 (22%) *
Hippocampus	164	20 (12%) *	19 (12%) *	14 (9%)	12 (7%)
vmPFC	105	1 (1%)	2 (2%)	3 (3%)	0 (0%)

**Table 2:** Neuronal responses per brain area that differed from background firing.

Area	Total # of Neurons	# Significant	Binomial *p*-Value	Adjusted *p*-Value
A	168	85 (34%)	8.9 × 10^−47^	1.6 × 10^−35^
ACC	188	74 (39%)	5.3 × 10^−46^	2.1 × 10^−45^
H	164	44 (27%)	2.6 × 10^−20^	3.5 × 10^−20^
vmPFC	105	16 (15%)	6.8 × 10^−5^	6.8 × 10^−5^

A = amygdala, ACC = anterior cingulate cortex, H = hippocampus, vmPFC = ventromedial prefrontal cortex.

**Table 3:** Average information for decoding race in single-neuron firing in each brain area.

Brain Area	Mutual Information (Bits)	# Neurons to Decode 2 Bits
A	0.022	90.1
ACC	0.021	94.4
H	0.021	94.5
vmPFC	0.156	128.0

A = amygdala, ACC = anterior cingulate cortex, H = hippocampus, vmPFC = ventromedial prefrontal cortex.

**Figure A1:**
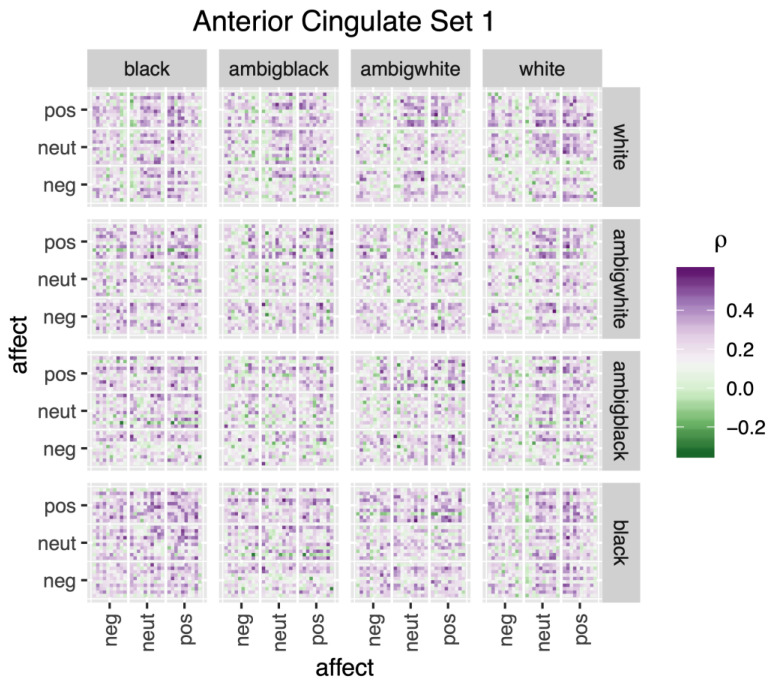
Representational similarity analysis of neural firing in the anterior cingulate cortex to face pairs, grouped first by race, then by affect. Each pixel represents the Pearson’s correlation coefficient for image pairs, with race and affect determined by the pixel’s position along the x and y axes (except for pixels with identical images in the pair, wherein the variance is shown). Stronger correlations are observed for the white-white and black-black pairs.

**Figure A2:**
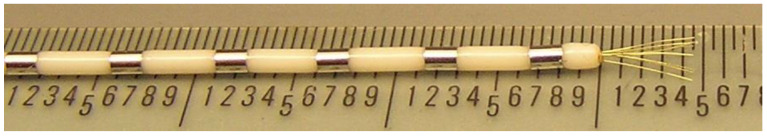
Image of clinical macroelectrode and microwire electrodes to scale. Numbers on the ruler indicate mm of length.

**Figure A3:**
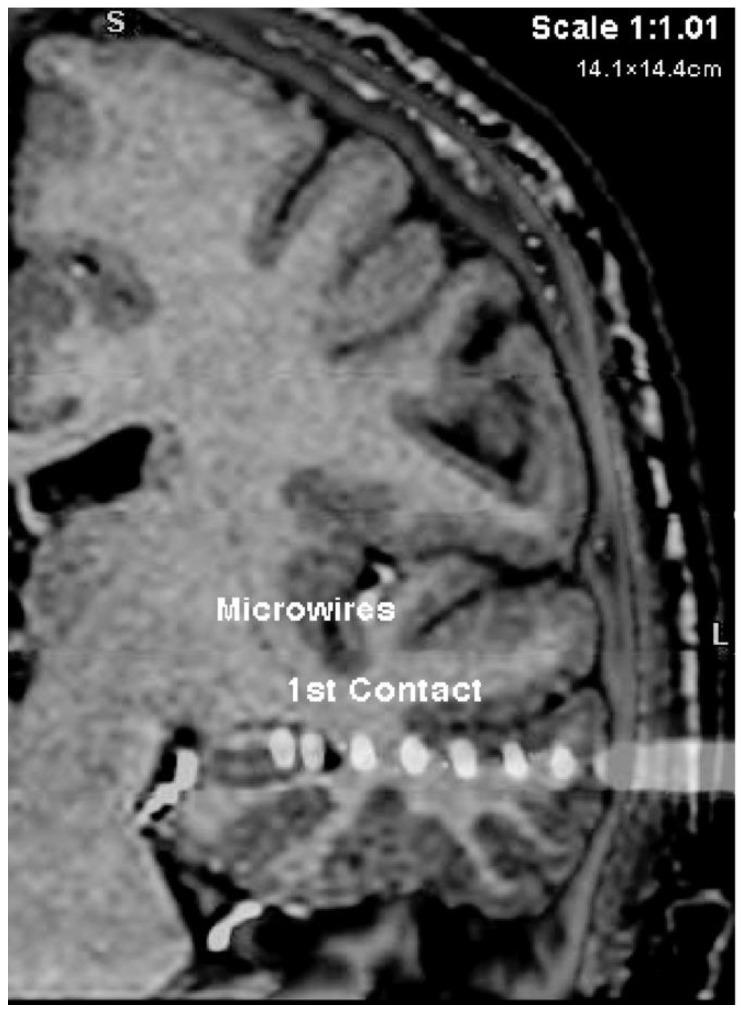
CT scan of the left hemisphere with macroelectrode and microwires implanted in the hippocampus.

## Data Availability

The data underlying this report are available at the Open Science Foundation at https://osf.io/nf7s8/ (accessed on 29 May 2022) as well as by request to info@neurtex.org (accessed on 29 May 2022).

## Significance Statement

Identifying the members of one’s social group is critical to social interactions. Several brain areas, such as the amygdala, are critical to this identification. Prior imaging studies have examined the hemodynamic correlates of race discriminations. Here we examined the activity of single neurons in the brains of epilepsy patients to determine how neurons fire as human subjects view face images belonging to different races. We report that neuronal firing rates in the amygdala, hippocampus, and prefrontal cortex all predicted race categories. These results suggest that race-selective neurons are broadly represented across brain regions and that such neurons respond to race-specific cues even when attention is directed toward different stimulus dimensions.

## Appendix A. Patient Details

**Table A1:** Characteristics and clinically identified seizure foci for all subjects (*n* = 14). Note the three subjects who were excluded from the primary analysis.

Age	Sex	Handedness	Ethnicity/Race	Brain Areas with Seizure Foci	Included in Primary Analysis
38	Female	Right	Caucasian White	Left amygdala, left hippocampus	No
54	Female	Right	Caucasian White	Left amygdala, right amygdala, right hippocampus	Yes
42	Male	Right	Caucasian White	Left amygdala, left hippocampus	Yes
35	Male	Left	Hispanic White	Right amygdala, right hippocampus	Yes
41	Male	Right	African-American Black	Left hippocampus, left frontal lobe	No
21	Female	Right	Caucasian White	Left amygdala, left hippocampus	Yes
24	Female	Right	Caucasian White	Right amygdala, right hippocampus, right frontal lobe	Yes
23	Male	Right	Caucasian White	Right amygdala, right hippocampus	Yes
54	Female	Right	Caucasian White	Left hippocampus	Yes
56	Female	Right	Caucasian White	Right occipital lobe	Yes
40	Female	Right	Hispanic White	Right amygdala, right hippocampus	Yes
54	Female	Right	Caucasian White	Right hippocampus	Yes
20	Male	Right	Caucasian White	Right temporal lobe	Yes
53	Female	Right	African-American Black	Right amygdala, right hippocampus, left amygdala, left hippocampus	No

## Appendix B. Extended Analyses

Appendix B contains tables and statistical notations excluding identified seizure onset zones, and analyses covering the entire sample excluding one patient whose session contained a technical error (the first patient in Table A1). For completeness, we also report tables for multi-unit analyses (MUA), which largely mirror those for the single-units (SUA) reported in the main text.

**Table A2:** Percentages of neurons significant for race category (relative to background firing) for each brain area, excluding all recordings from identified seizure onset zones. Values with asterisks denote significant proportions in binomial tests (*p* < 0.05), after correction for multiple comparisons.

	# Neurons	Black	Ambiguous Black	Ambiguous White	White
Amygdala	101	26 (26%) *	22 (22%) *	28 (27%) *	30 (30%) *
ACC	188	43 (23%) *	35 (19%) *	36 (19%) *	41 (22%) *
Hippocampus	84	14 (17%) *	12 (14%) *	9 (11%) *	7 (8%)
vmPFC	105	1 (1%)	2 (2%)	3 (3%)	0 (0%)

**Table A3:** Neurons significant for specific race categories (relative to background firing) by side and brain area (SUA), across all patients (*n* = 13), as indicated by multinomial logistic regression models. Values with asterisks denote significant proportions in binomial tests (*p* < 0.05), after correction for multiple comparisons.

Area	Total # of Neurons	Sig. for White	Sig. for Black	Sig. for Ambiguous White	Sig. for Ambiguous Black
LA	128	30 (23%) *	27 (21%) *	30 (23%) *	24 (19%) *
RA	89	7 (8%)	3 (3%)	3 (3%)	4 (4%)
LACC	164	24 (15%) *	28 (17%) *	20 (12%) *	21 (13%) *
RACC	92	20 (22%) *	17 (18%) *	18 (20%) *	17 (18%) *
LH	143	10 (7%)	12 (8%)	7 (5%)	15 (10%) *
RH	99	6 (6%)	10 (10%)	10 (10%)	7 (7%)
LvmPFC	87	1 (1%)	1 (1%)	4 (5%)	0 (0%)
RvmPFC	79	0 (0%)	0 (0%)	0 (0%)	2 (3%)

A = amygdala, ACC = anterior cingulate cortex, H = hippocampus, PFC = ventromedial prefrontal cortex.

**Table A4:** Significant neuronal responses (relative to background firing) by side and brain area (SUA) among all subjects (*n* = 13), as indicated by the changes from background test.

Side/Area	Total # of Neurons	# Significant	Adjusted Binomial *p*-Value
LA	128	56 (44%)	2.6 × 10−37
RA	89	17 (19%)	2.4 × 10−6
LACC	164	56 (34%)	8.5 × 10−31
RACC	92	29 (32%)	1.6 × 10−15
LH	143	29 (20%)	1.8 × 10−10
RH	99	27 (27%)	6.0 × 10−13
LvmPFC	87	16 (18%)	6.9 × 10−6
RvmPFC	79	7 (9%)	1.0 × 10−1

A = amygdala, ACC = anterior cingulate cortex, H = hippocampus, vmPFC = ventromedial prefrontal cortex.

**Table A5:** Clusters (MUA) significant for specific race categories (relative to background firing) by side and brain area across all patients (*n* = 13), as indicated by multinomial logistic regression models. Values with asterisks denote significant proportions in binomial tests (*p* < 0.05), after correction for multiple comparisons.

Area	Total # of Clusters	Sig. for White	Sig. for Black	Sig. for Ambiguous White	Sig. for Ambiguous Black
LA	248	34 (14%) *	46 (19%) *	29 (12%) *	37 (15%) *
RA	173	5 (3%)	5 (3%)	1 (1%)	7 (4%)
LACC	207	43 (21%) *	31 (15%) *	28 (14%) *	22 (11%) *
RACC	213	32 (15%) *	30 (14%) *	34 (16%) *	34 (16%) *
LH	226	13 (6%)	10 (4%)	5 (2%)	6 (3%)
RH	198	7 (4%)	7 (4%)	6 (3%)	6 (3%)
LvmPFC	217	11 (5%)	13 (6%)	11 (5%)	10 (5%)
RvmPFC	197	4 (2%)	1 (1%)	1 (1%)	2 (1%)

A = amygdala, ACC = anterior cingulate cortex, H = hippocampus, vmPFC = ventromedial prefrontal cortex.

**Table A6:** Reliable cluster responses (MUA) by side and brain area across all patients (*n* = 13), as indicated by the change from background test.

Side/Area	Total # of Clusters	# Significant	Adjusted Binomial *p*-Value
LA	248	77 (31%)	1.3 × 10−38
RA	173	32 (18%)	2.6 × 10−10
LACC	207	74 (36%)	1.2 × 10−41
RACC	213	58 (27%)	3.4 × 10−26
LH	226	38 (17%)	1.3 × 10−10
RH	198	21 (11%)	1.7 × 10−3
LvmPFC	217	29 (13%)	2.2 × 10−6
RvmPFC	197	12 (6%)	2.8 × 10−1

A = amygdala, ACC = anterior cingulate cortex, H = hippocampus, vmPFC = ventromedial prefrontal cortex.

**Representational Similarity Analysis.** As a final step, we conducted representational similarity analyses (RSA) [79,80,81] to help visualize any joint effects of stimulus race and affect. To perform RSA, responses for each neuron to each image were first normalized to the average background firing rate of that neuron in the interval from 1000–200 ms prior to stimulus presentation (this interval was chosen to mirror that used for computing responses) by subtracting the mean background spike count and dividing by the standard deviation of the background spike counts. Next, Pearson’s correlations between the normalized responses for all image pairs were computed. For pairs consisting of a stimulus with itself, the variance of the observations, rather than the correlation (which would always equal one), was used. Finally, these values were plotted in two different orderings. Of primary interest (see Figure A1), pairs were first ordered by affect and race, such that the outer larger blocks showed effects of race, averaged across affects. Figure A1 shows the average correlations of neural responses in the ACC, ordered first by race categories and then by emotions for faces in the stimulus set 1. A strong response to race would manifest as stronger correlations in major blocks along the diagonal, relative to the off-diagonal blocks, as shown. This figure also shows a tendency for stronger correlations when White-White or Black-Black face pairs were compared. Both appear as a pattern of less purple in the four central major blocks. Similar (although less visually apparent) patterns were observed for the hippocampus and amygdala, and for the stimulus set 2.

To statistically assess the apparent pattern of stronger correlations in the main diagonal blocks, we used the technique from [82,83], a permutation test [54] based on the Wilcoxon rank-sum test statistic [33]. For each brain area, we computed the rank-sum statistic comparing all correlation values (in both stimulus sets) in the off-main versus the on-main diagonal blocks (excluding the variances). We then randomly permuted the race labels of the second image per pair and re-computed the rank-sum statistics. This permutation was performed 1000 times; the rank of the original (non-permuted) test statistic was used to obtain the corresponding *p*-value. The pattern of stronger correlations in the main diagonal blocks was significant in all four brain areas, indicating that race-responsive neurons were reasonably consistent in their response profiles. Admittedly, the statistical evidence is challenging to visually appreciate in Figure A1. Since race variations were orthogonal our experimental task, we posit that race-sensitive neural responses were attenuated.
